# Neuroimaging in normal pressure hydrocephalus

**DOI:** 10.1590/1980-57642015DN94000350

**Published:** 2015

**Authors:** Benito Pereira Damasceno

**Affiliations:** 1 MD, PhD, Department of Neurology, Medical School, University of Campinas (UNICAMP), Campinas SP, Brazil.

**Keywords:** normal pressure hydrocephalus, neuroimaging, magnetic resonance, cerebrospinal fluid tap test, shunt surgery, hidrocefalia de pressão normal, neuroimagem, ressonância magnética, teste da punção lombar, cirurgia de derivação liquórica

## Abstract

Normal pressure hydrocephalus (NPH) is a syndrome characterized by the triad of gait disturbance, mental deterioration and urinary incontinence, associated with ventriculomegaly and normal cerebrospinal fluid (CSF) pressure. The clinical presentation (triad) may be atypical or incomplete, or mimicked by other diseases, hence the need for supplementary tests, particularly to predict postsurgical outcome, such as CSF tap-tests and computed tomography (CT) or magnetic resonance imaging (MRI). The CSF tap-test, especially the 3 to 5 days continuous external lumbar drainage of at least 150 ml/day, is the only procedure that simulates the effect of definitive shunt surgery, with high sensitivity (50-100%) and high positive predictive value (80-100%). According to international guidelines, the following are CT or MRI signs decisive for NPH diagnosis and selection of shunt-responsive patients: ventricular enlargement disproportionate to cerebral atrophy (Evans index >0.3), and associated ballooning of frontal horns; periventricular hyperintensities; corpus callosum thinning and elevation, with callosal angle between 40º and 90º; widening of temporal horns not fully explained by hippocampal atrophy; and aqueductal or fourth ventricular flow void; enlarged Sylvian fissures and basal cistern, and narrowing of sulci and subarachnoid spaces over the high convexity and midline surface of the brain. On the other hand, other imaging methods such as radionuclide cisternography, SPECT, PET, and also DTI or resting-state functional MRI, although suitable for NPH diagnosis, do not yet provide improved accuracy for identifying shunt-responsive cases.

## Abstract (pt)

A hidrocefalia de pressão normal (HPN) é uma síndrome caracterizada por alteração da marcha, transtorno mental-cognitivo e incontinência urinária, associados a ventriculomegalia e pressão liquórica normal. A apresentação clínica (tríade) pode ser atípica ou incompleta, ou pode ser mimetizada por outras doenças, daí a necessidade de testes suplementares, principalmente para predição do resultado cirúrgico, tais como teste da punção lombar e tomografia computadorizada (TC) ou ressonância magnética (MR) de crânio. O teste da punção liquórica lombar, especialmente a drenagem externa contínua (≥150 ml/dia, por 3 a 5 dias), é o único método que simula o efeito da cirurgia, com alta sensibilidade (50-100%) e alto valor preditivo positivo (80-100%). Consensos internacionais consideram os seguintes achados da TC ou RM como decisivos para o diagnóstico de HPN e a seleção de pacientes bons respondedores à cirurgia: dilatação ventricular desproporcional em relação ao grau de atrofia cerebral (índice de Evans >0.3), associada a arredondamento dos cornos frontais; hipersinal difuso periventricular; adelgaçamento e elevação do corpo caloso, com ângulo do corpo caloso entre 40º e 90º; dilatação dos cornos temporais não explicada por atrofia hipocampal; sinal do fluxo vazio no aqueduto e quarto ventrículo; dilatação das fissuras Sylvianas e cisterna basal, e estreitamento ou apagamento dos sulcos e espaços subaracnoides nas superfícies cerebrais da convexidade alta e linha média. Por outro lado, a cisternografia isotópica, SPECT, PET, e mesmo técnicas mais modernas de RM funcional e tensor de difusão, embora compatíveis com o diagnóstico de HPN, não melhoram a acurácia na identificação de casos responsivos à cirurgia.

## INTRODUCTION

Normal pressure hydrocephalus (NPH) is a syndrome characterized by the triad of gait disturbance, mental deterioration and urinary incontinence, which are associated with enlargement of the ventricular system and normal cerebrospinal fluid (CSF) pressure. In NPH, CSF pressure may be normal at one spinal tap, but episodes of increased CSF pressure can occur, and hence NPH is also termed "intermittent pressure hydrocephalus". It is caused by excessive accumulation of CSF in the ventricular system due to an impairment of its flow distally to the fourth ventricle ("communicating" hydrocephalus). About 50% of cases with communicating NPH have a known cause (secondary or symptomatic NPH, or SNPH), such as meningitis, subarachnoid hemorrhage, or cranial trauma, while the remaining 50% of cases are idiopathic (INPH), usually presenting in the 7th decade of life. Epidemiological data on NPH incidence and prevalence are scarce, but surveys in Germany, Norway, Sweden and Japan have estimated the annual incidence of INPH to be between 1.8/100,000 and 5.5/100,000 inhabitants, with a prevalence ranging from 0.2% to 2.9% among individuals aged 65 years or older,^1-4^ and that it is the cause of dementia in up to 6% of all dementia cases. This review was based on a PubMed literature search from 1996 to date.

## DIAGNOSIS

The diagnosis of NPH is based on the following criteria:

[1] a history of gait disturbance, progressive mental deterioration, and urinary urgency or incontinence;
[2] hydrocephalus, defined as Evans' ratio >0.30on computed tomography (CT) or magnetic resonance (MR) imaging; and
[3] a CSF opening pressure (appropriately measured) of <24 cm of water.

Differential diagnostic difficulties may arise when the clinical manifestations of the triad are atypical or incomplete, or when they are mimicked by other diseases. Indeed, many other conditions may cause the complete triad, such as vascular dementia, parkinsonism, Lewy body disease, corticobasal degeneration, progressive supranuclear palsy, multiple system atrophy, neurosyphilis, medication side effects; or in combination with other diseases, particularly cerebrovascular and Alzheimer's disease, which are present in up to 75% of patients with INPH.^5^

## NEUROIMAGING AND COMPLEMENTARY PROGNOSTIC TESTS

Shunt surgery can improve all NPH symptoms and quality of life in up to 80% of properly selected cases, but has complications rates (35-52%) that dissuade us from shunting every suspected case. Surgical decision may be difficult and yield varying results, particularly in the elderly with comorbidities or vascular or degenerative brain diseases, which can mimic or worsen NPH symptoms. Therefore, in order to improve diagnosis and management of NPH cases, complementary tests have to be used, especially neuroimaging and CSF tap tests.

Radionuclide cisternography (RC), intracranial pressure monitoring (ICP) and lumbar infusion tests can show CSF dynamics malfunction, but none are able to confirm whether the patient will benefit from surgery.^6,7^ A 'positive' RC (with ventricular reflux and convexity block) can be seen in other dementia disorders and even in healthy subjects, thus having questionable predictive value. Most clinicians suggest it should no longer be performed,^8^ and the International INPH Guidelines^9^ have not included it as an option, since it does not improve the diagnostic accuracy of identifying shunt-responsive cases.

The CSF tap test (CSF-TT) consists of quantitative testing of gait and cognitive functions before and after the drainage of 40-50 ml lumbar CSF. It is the only procedure that can temporarily simulate the effect of a definitive shunt, and can predict not only the outcome of surgery but also the degree of improvement.^10,11^ Since the one tap CSF-TT has low sensitivity (26-61%), a "negative" result cannot be used to exclude patients from surgery. In such cases, the alternative is a repeated CSF-TT (RTT) performed on three consecutive days with a minimum of 30 to 40 ml CSF removed; or continuous external lumbar drainage (ELD) for 3 to 5 days, with a minimum of 150 ml CSF drained daily. Due to ELD's high sensitivity (50-100%) and high positive predictive value (80-100%), it is considered by the 2005 International INPH Guidelines the most effective test for identifying shunt-responsive cases, even though it requires hospital admission and is associated with higher complication rates (meningitis, subdural hematoma, nerve root inflammation).

In NPH, computed tomography (CT) and magnetic resonance imaging (MRI) show ventricular enlargement disproportionate to cerebral atrophy, with associated ballooning of frontal horns, periventricular hyperintensities, thinning and elevation of the corpus callosum, and widening of temporal horns without evidence of hippocampal atrophy. Although diagnosis can be made on the basis of CT findings alone, MRI is more accurate for disclosing associated pathologies (such as cerebrovascular disease) and also for detecting NPH typical signs of prognostic value, besides avoiding exposure to ionizing radiation.

The International Guidelines have recommended the following key imaging features for diagnosis of INPH and selection of shunt-responsive patients:

1. Ventricular enlargement not entirely attributable to cerebral atrophy or congenital enlargement (Evans index >0.3).
2. No macroscopic obstruction to CSF flow.
3. At least one of the following supportive features:
  - Enlargement of the temporal horns of the lateral ventricles not entirely attributable to hippocampus atrophy;
  - Callosal angle of 40º or greater;
  - Evidence of altered brain water content, including periventricular signal changes on CT and MRI not attributable to microvascular ischemic changes or demyelination;
  - An aqueductal or fourth ventricular flow void on MRI.

The Japanese INPH Guidelines^12^ also include the following key imaging features:

(1) enlarged Sylvian fissures and basal cistern; and
(2) narrowing of the sulci and subarachnoid spaces over the high convexity and midline surface of the brain. Unlike the International Guidelines, periventricular changes are not considered essential.

Other neuroimaging methods have also been used in NPH. Single photon emission computed tomography (SPECT) and positron emission tomography (PET) can show reduction of cerebral blood flow and metabolism, mainly in frontobasal and anterior periventricular regions, even with improvement of regional cerebral metabolic rate of glucose in shunt responsive patients.^13^ Newer MRI techniques such as DTI^14^ and resting-state functional MRI^15^ have also been studied in NPH allowing the possibility of detecting biomarkers and improving NPH diagnosis. However, the diagnostic and prognostic value of all these neuroimaging techniques is not well established and they are not part of the routine selection procedures for shunt surgery.

**Figure 1:**
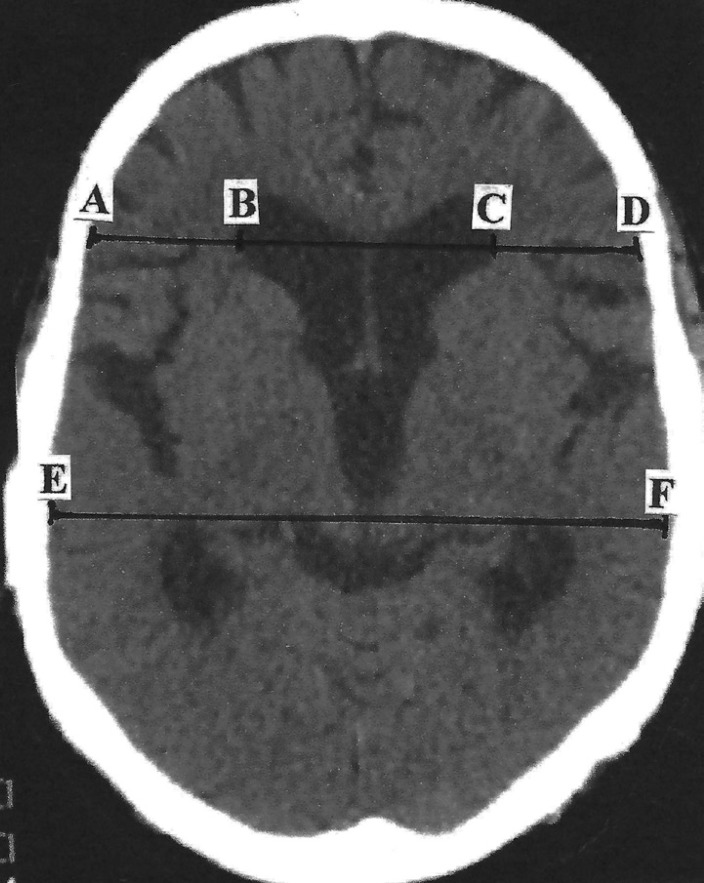
Axial CT slice of the brain in a patient with NPH. The Evans index can be measured by dividing the maximal width of the frontal horns [B-C] by the maximal width of the inner table of the cranium at the level of the frontal horns [A-D]; or by an equivalent measure, such as by dividing the diameter of the frontal horns [B-C] by the widest brain diameter [E-F].

Ventricular enlargement can be measured by the Evans índex,^16^ which is the ratio between the maximal width of the frontal horns and the maximal width of the inner table of the cranium at the level of the frontal horns; or by an equivalent measure, such as by dividing the diameter of the frontal horns by the widest brain diameter (Figure 1).

The reliability of the Evans index has been questioned by some studies using modern brain imaging techniques (FreeSurfer) for ventricular volumetric analysis.^17^ However, a more recent study^18^ has addressed this issue and showed that the Evans index and other linear measurements (frontal-occipital horn ratio, third ventricular width, and callosal angle at the level of the posterior commissure) reliably determine ventricular enlargement, without the need for expensive, time-intensive and technically challenging computer software, unavailable in many healthcare services.

Periventricular signal changes may be associated with subcortical vascular encephalopathy (also with lacunar infarctions) in NPH, but this does not predict poor surgical outcome and should not exclude patients from shunting.^5,19^

**Figure 2:**
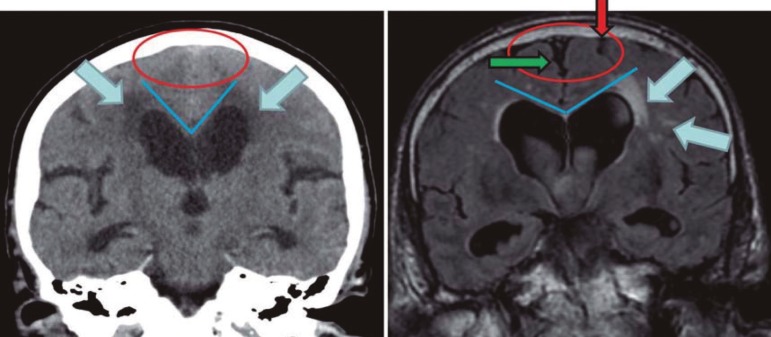
Coronal head CT (left) and MRI (right) at the level of the posterior commissure: in the left image, the CSF spaces over the convexity near the vertex are narrowed ("tight convexity", red circle), as are the medial cisterns (red circle) - these are typical findings of NPH. On the right image, however, the CSF spaces over the convexity near the vertex (red arrow) and the medial cisterns (green arrow) are widened, a finding consistent with brain atrophy. The blue lines in both images indicate the callosal angle: an angle less than 90º is typical of NPH (left image), while an angle greater than 90º is typical of brain atrophy (right image). The blue arrows indicate periventricular signal alterations. The unilateral occurrence of these alterations (right image) suggests they are probably due to vascular encephalopathy. The abnormalities seen in the left image may well represent transependymal CSF diapedesis due to NPH. (From Kiefer & Unterberg, Dtsch Arztebl Int, 2012, with permission).

**Figure 3:**
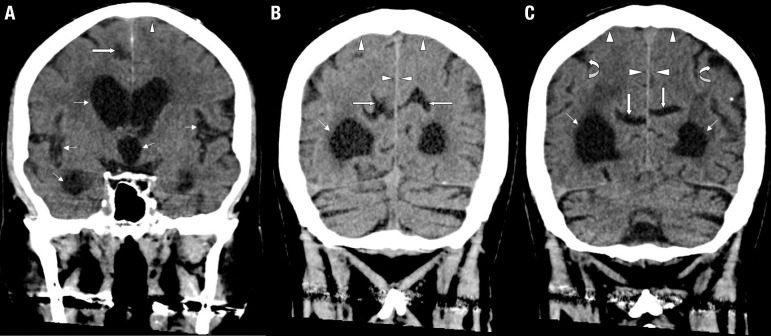
Coronal head CT of a 73-year-old man with idiopathic NPH. [A, B and C] show disproportionately enlarged ventricles with periventricular hypointense signal alterations, and expanded sylvian fissure and insular cisterns (thin black arrows), narrowed sulci and subarachnoid spaces at the high convexity near the vertex and midline (white arrow heads), as well as focally dilated sulci over the convexity (curved white arrows) and medial surfaces (straight white arrows).

In typical NPH cases, the ventricles are disproportionately more dilated than the cortical sulci, which are narrow or obliterated at the high convexity and midline, with local narrowing of the subarachnoid space surrounding the outer brain surface, as can be seen in a MRI coronal section at the level of the posterior commissure (Figure 2 and 3). In this context, the presence of enlarged ventricles associated with large basal cisterns and Sylvian fissures, and also focally dilated sulci, should not be misinterpreted as cerebral atrophy. On the contrary, these findings tend to support rather than exclude the diagnosis of shunt-responsive NPH.^20,21^ In normal aging and degenerative diseases (Alzheimer's, Pick's), the thinning of the gyri and the corresponding dilation of the sulci are more generalized, occurring to a similar degree in the affected brain regions.^22^

Callosal angle (CA) as well as temporal horns and hippocampus are best evaluated with coronal MRI. CA is the angle between the lateral ventricles (Figure 2), and is typical of NPH when between 40º and 90º. CA greater than 90º suggests brain atrophy,^5^ as occurs in degenerative diseases such as Alzheimer's and Lewy body dementia.^23^ In a recent study, shunt-responders had a significantly smaller mean preoperative CA compared with non-responders (59% versus 68%), with a cut-off value of 63% providing the best prognostic accuracy.^24^ Another consistent finding in NPH, best recognized with mid-sagittal MRI, is callosal upward elevation, stretching and thinning, usually showing total or partial recovery after shunting.^25^

Wide temporal horns are typical of NPH and represent significant predictors of a positive shunt outcome,^26^ although narrow temporal horns are also compatible with NPH diagnosis.^27^ Evaluation of temporal horns and hippocampal/perihippocampal structures may distinguish hydrocephalic enlargement of the ventricle, as occurs in NPH (with obliteration of perihippocampal sulcal markings) from ventriculomegaly secondary to cerebral atrophy (which may expose sulcal markings in this region).^28^ Measurement of the hippocampus volume may be of diagnostic and prognostic value in cases of suspected NPH versus Alzheimer's disease (AD), in which the hippocampus is significantly smaller.^29^ Diffusion tensor imaging (DTI) of the fornix may also differentiate NPH from AD. Fornix volume, cross-sectional area, and fractional anisotropy are smaller both in NPH and in AD relative to controls. Fornix length, however, is significantly greater in NPH than in controls yet not altered in AD, probably explained by mechanical stretching due to lateral ventricular dilation and corpus callosum deformation in NPH and by degeneration secondary to hippocampal atrophy in AD.^30^

Aqueductal flow void is a loss (increased hypointensity) of signal seen within the aqueduct and neighbouring third and fourth ventricles, particularly on T2-weighted MRI images. The signal loss is usually increased in states of hyperdynamic CSF motion, with higher velocities, turbulent and accelerated flow, which occur mainly in passages with smaller cross-sectional areas such as the aqueduct. In NPH there is greater outflow of CSF through the aqueduct with subsequent increase in signal loss (void sign). The first studies^31,32^ showed correlation between void sign in the aqueduct and shunt results, while others^33^ did not, with the same frequency of void sign occurring in NPH patients and healthy controls.

Further advances in CSF flow imaging have enabled quantification by means of cine phase-contrast MRI (PC-MRI) throughout the cardiac cycle. With this technique, the slice is positioned on an angled axial plane perpendicular to the aqueduct using higher spatial resolution MRI because of the small size of this structure, as well as short TR to achieve adequate temporal resolution.^34,35^ The aqueductal CSF stroke volume (ACSV, defined as the average of the volume flowing down during cardiac systole and up during diastole) is then calculated. Initial studies^36^ found that NPH patients who responded to shunting had aqueductal stroke volume > 42 µL or at least twice the ACSV of healthy elderly subjects, while others did not.^37,38^ For this reason, a single PC-MRI measurement of stroke volume cannot reliably predict which patients will improve after shunting.^35^ In one study, 14% of patients who did not improve after a high-volume lumbar tap test had significantly higher aqueductal CSF flow rates than patients who improved.^38^

On the other hand, the combination of PC-MRI with the tap test by measuring the peak CSF flow velocity at the level of the aqueduct, before and after lumbar CSF drainage, has been shown to be a sensitive method to support the diagnosis of NPH and select patients who are likely to benefit (or not) from shunt surgery.^34,39^

Thus, the findings of aqueductal CSF flow void alone, or of a single PC-MRI measurement, have long been observed even in healthy persons, and cannot safely support NPH diagnosis or postsurgical prognosis.

More recent MRI techniques for CSF flow study, such as time-spatial labeling inversion pulse (Time-SLIP),^40^ have been introduced, but their predictive value for selecting shunt-responsive NPH patients needs to be further investigated with more extensive studies.^35^

In conclusion, NPH is a treatable cause of dementia, with the best shunting results occurring when the CSF tap test is positive and CT or MRI show signs of high diagnostic and predictive value, such as: ventricular enlargement disproportionate to cerebral atrophy (Evans index > 0.3) with ballooning of frontal horns; periventricular hyperintensities; corpus callosum thinning and elevation, callosal angle between 40º and 90º; widening of temporal horns not entirely explained by hippocampal atrophy; aqueductal or fourth ventricular flow void; enlarged Sylvian fissures and basal cistern, and narrowing of sulci and subarachnoid spaces over the high convexity and midline surface of the brain.
